# Supplementary figures and images for: Recruitment of Polo-like kinase couples synapsis to meiotic progression via inactivation of CHK-2
Source: eLife. 2023 Jan 26;12:e84492. doi: 10.7554/eLife.84492 (PMC9998088; doi:10.7554/eLife.84492)

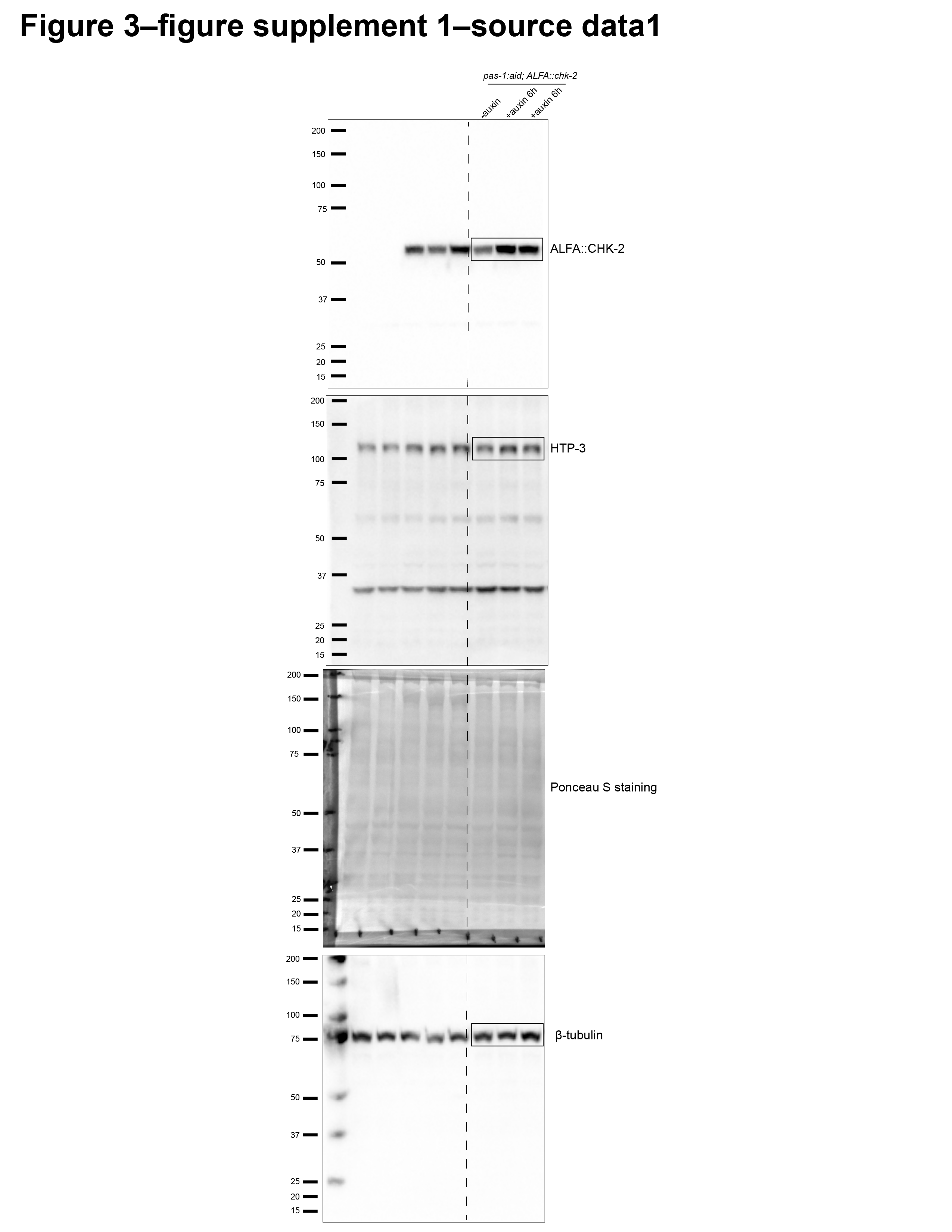

Supplement: Figure 3—figure supplement 1—source data 1. [file elife-84492-fig3-figsupp1-data1.zip › raw images.png]

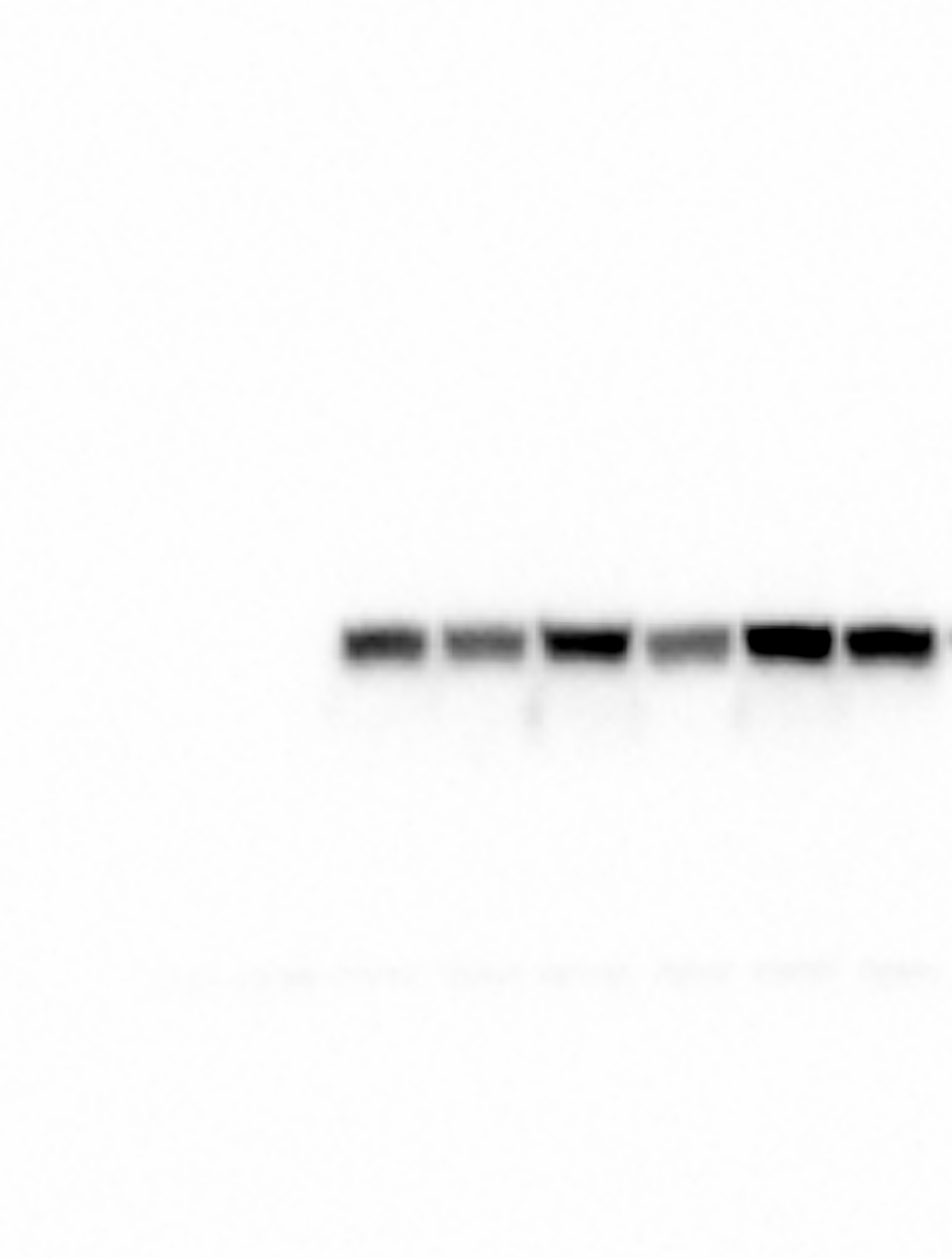

Supplement: Figure 3—figure supplement 1—source data 1. [file elife-84492-fig3-figsupp1-data1.zip › raw images/ALFA blot.tif]

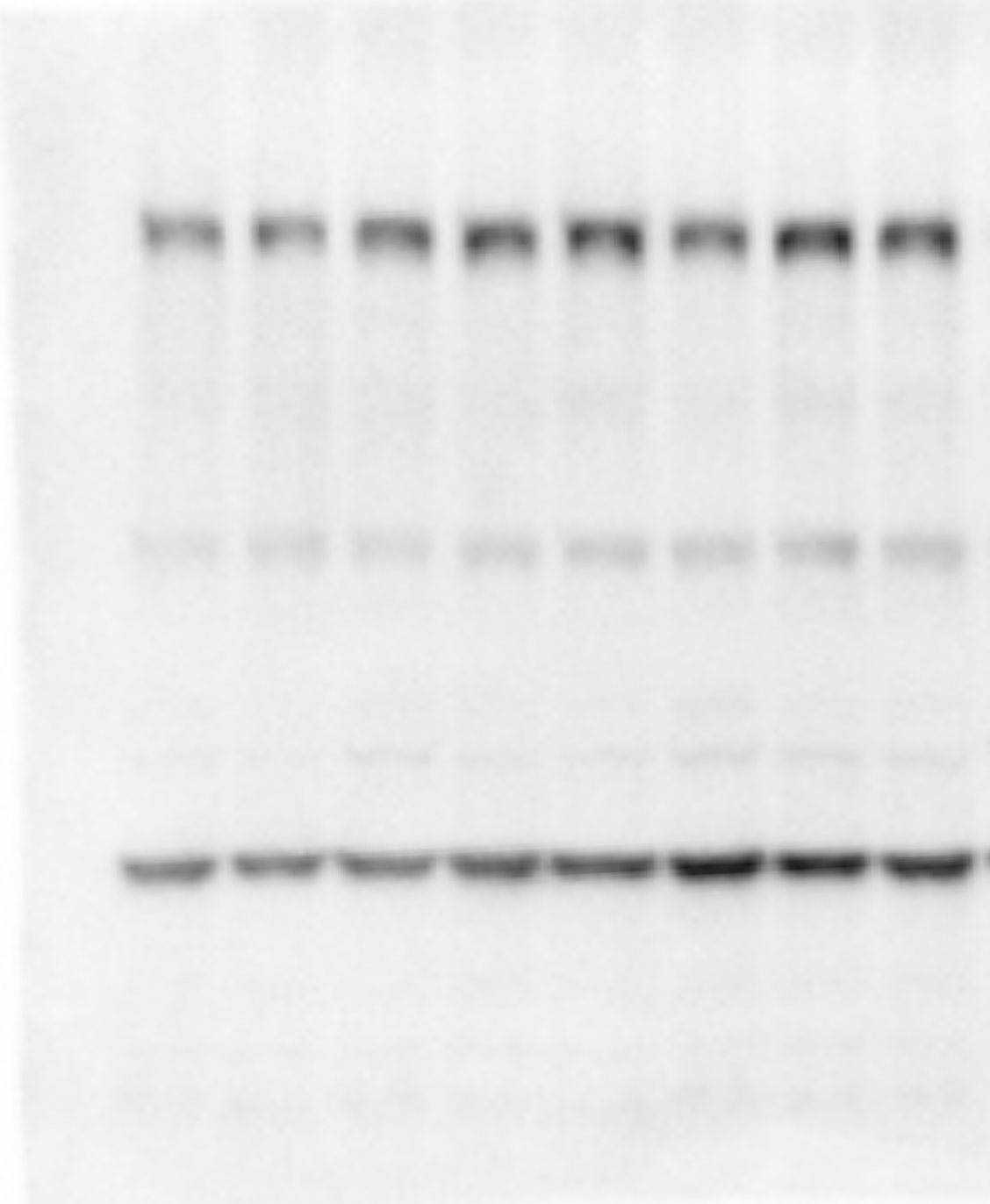

Supplement: Figure 3—figure supplement 1—source data 1. [file elife-84492-fig3-figsupp1-data1.zip › raw images/HTP3 blot.tif]

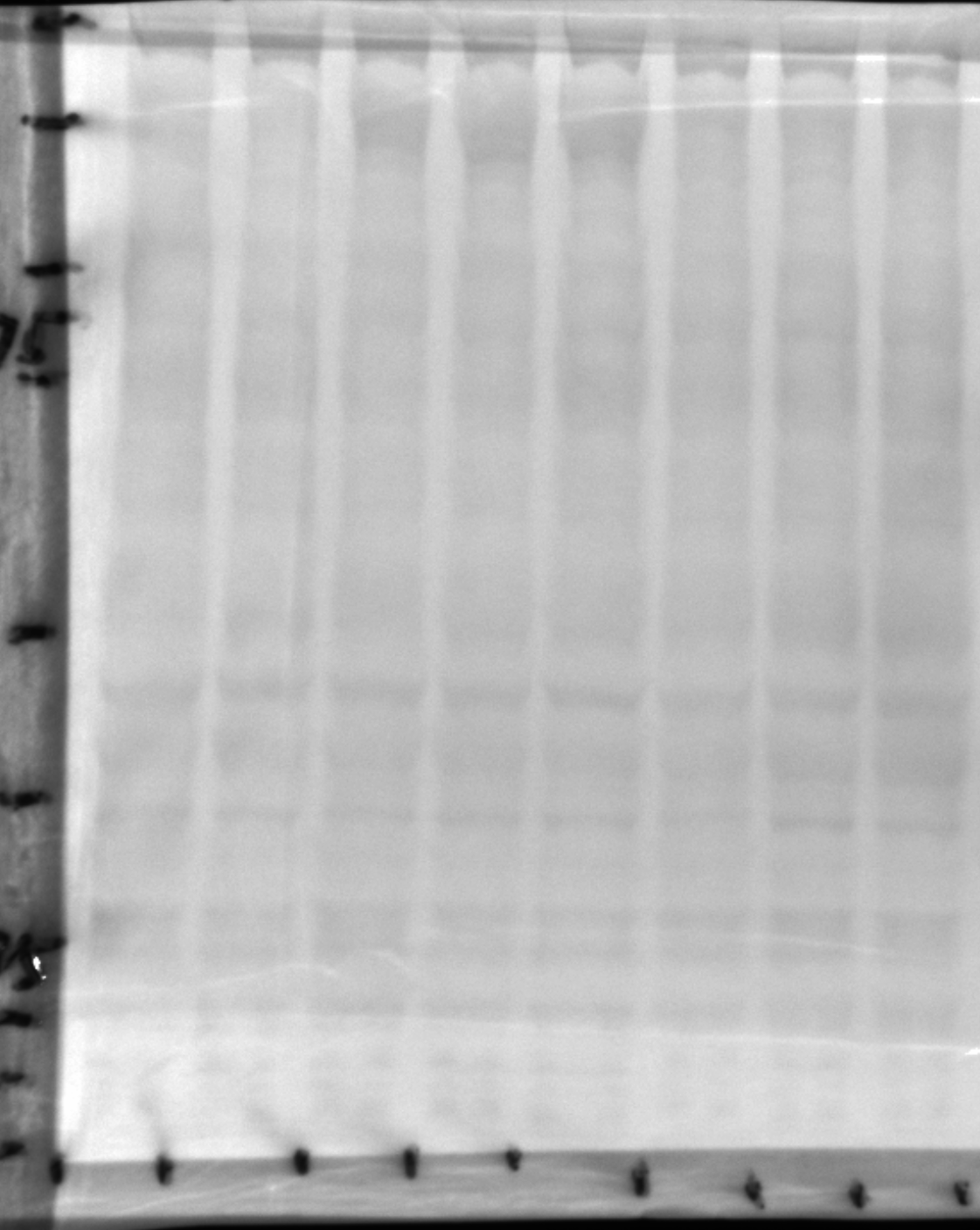

Supplement: Figure 3—figure supplement 1—source data 1. [file elife-84492-fig3-figsupp1-data1.zip › raw images/Ponceau S.tif]

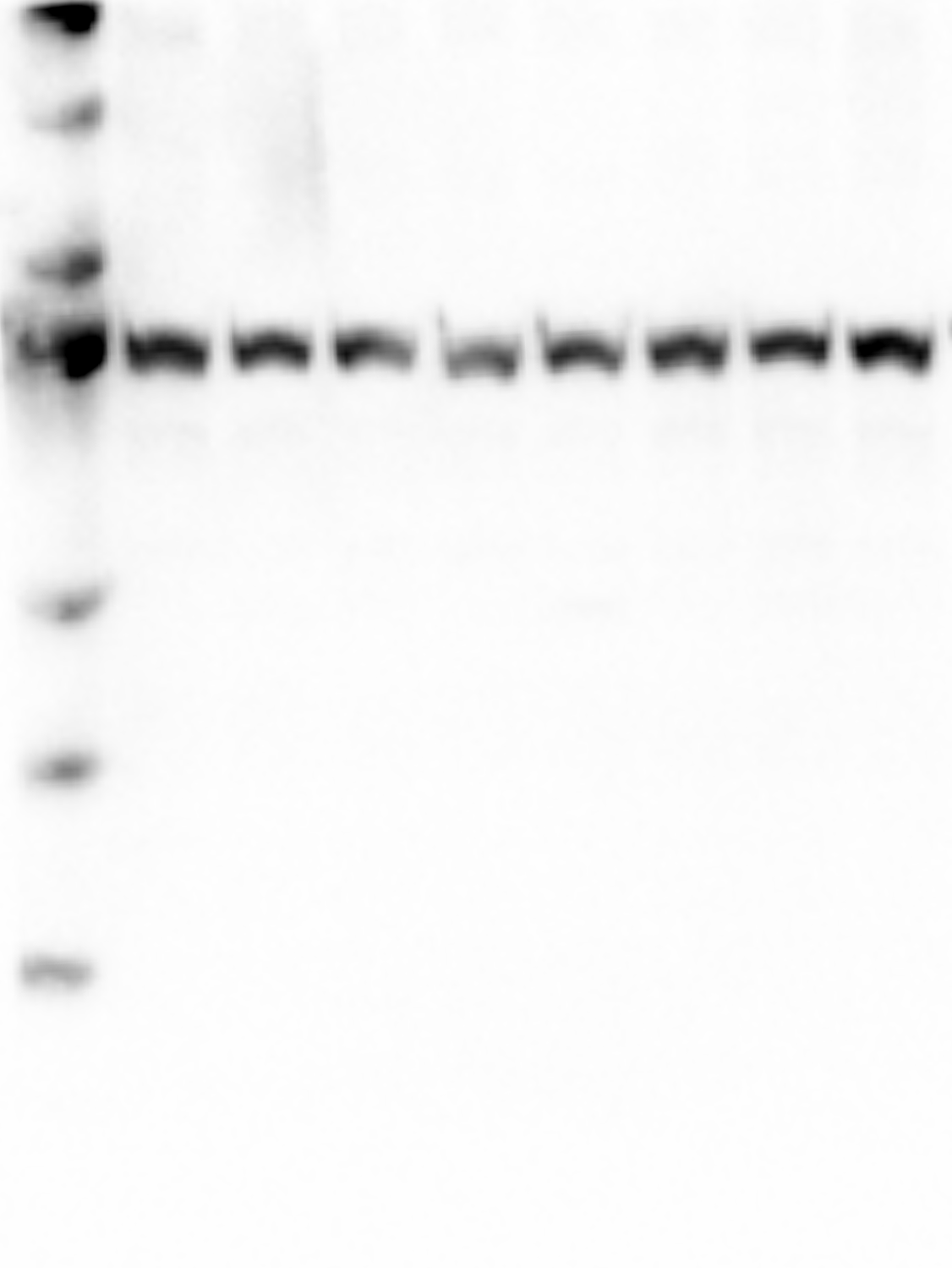

Supplement: Figure 3—figure supplement 1—source data 1. [file elife-84492-fig3-figsupp1-data1.zip › raw images/tubulin blot.tif]

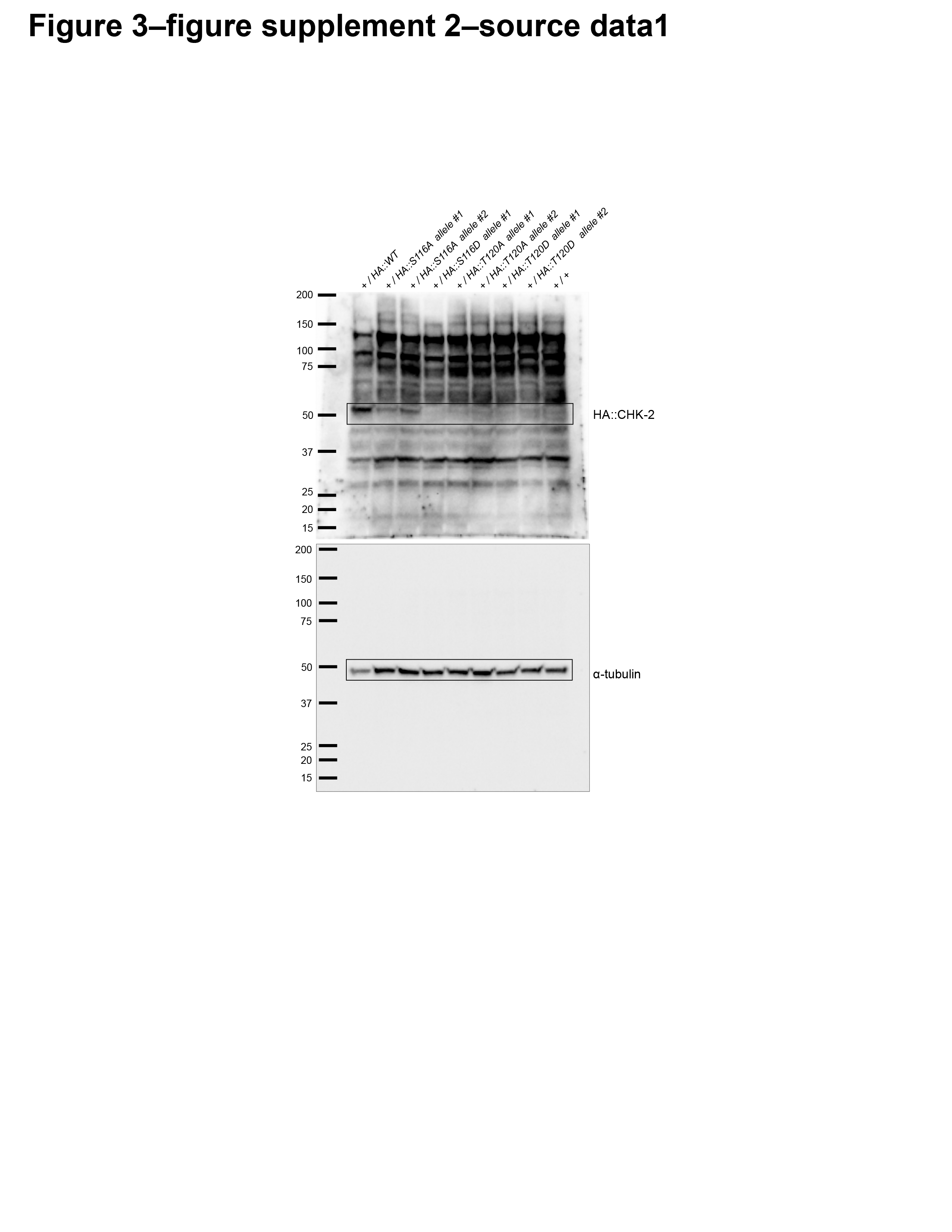

Supplement: Figure 3—figure supplement 2—source data 1. [file elife-84492-fig3-figsupp2-data1.zip › raw images.png]

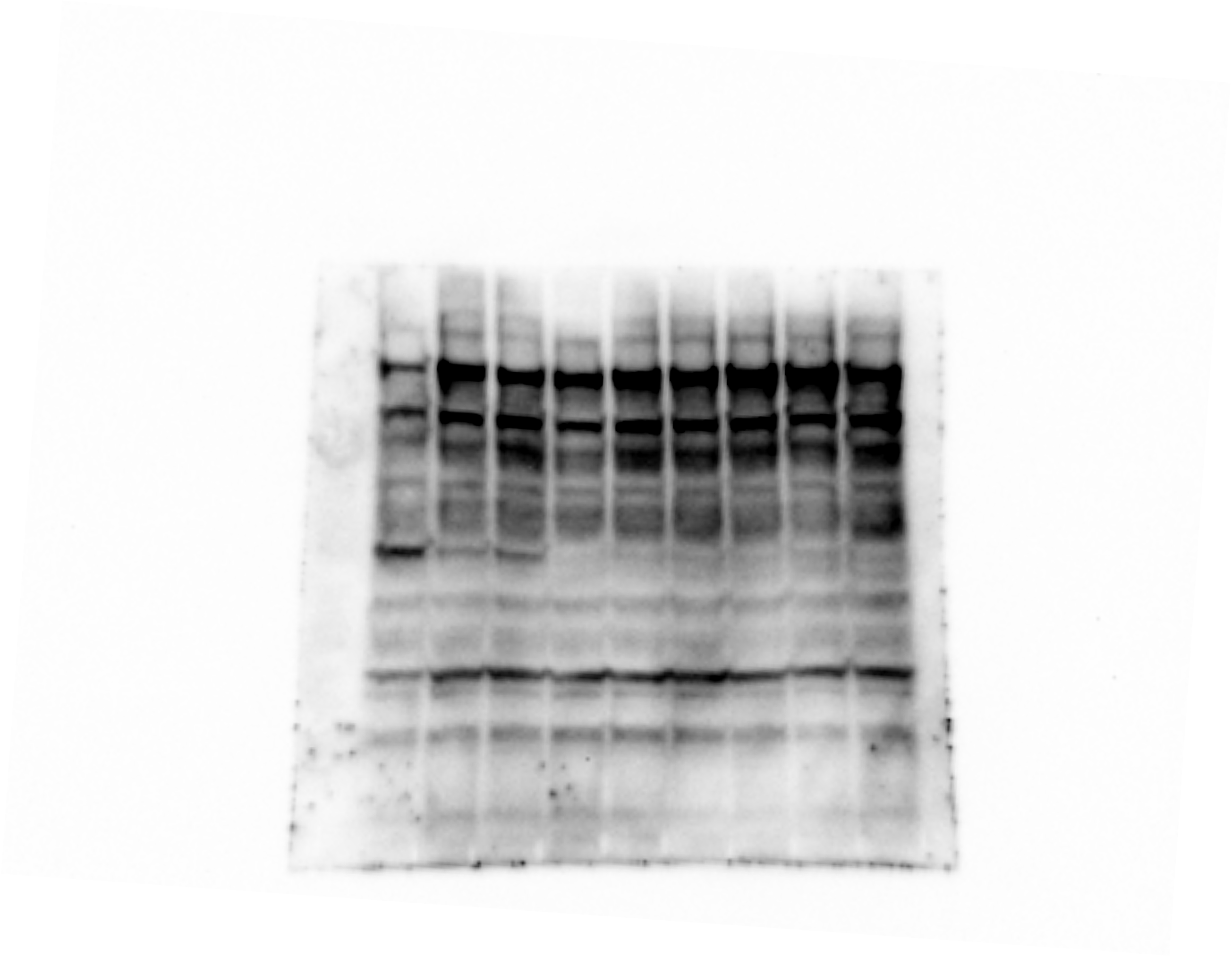

Supplement: Figure 3—figure supplement 2—source data 1. [file elife-84492-fig3-figsupp2-data1.zip › raw images/HA blot.tif]

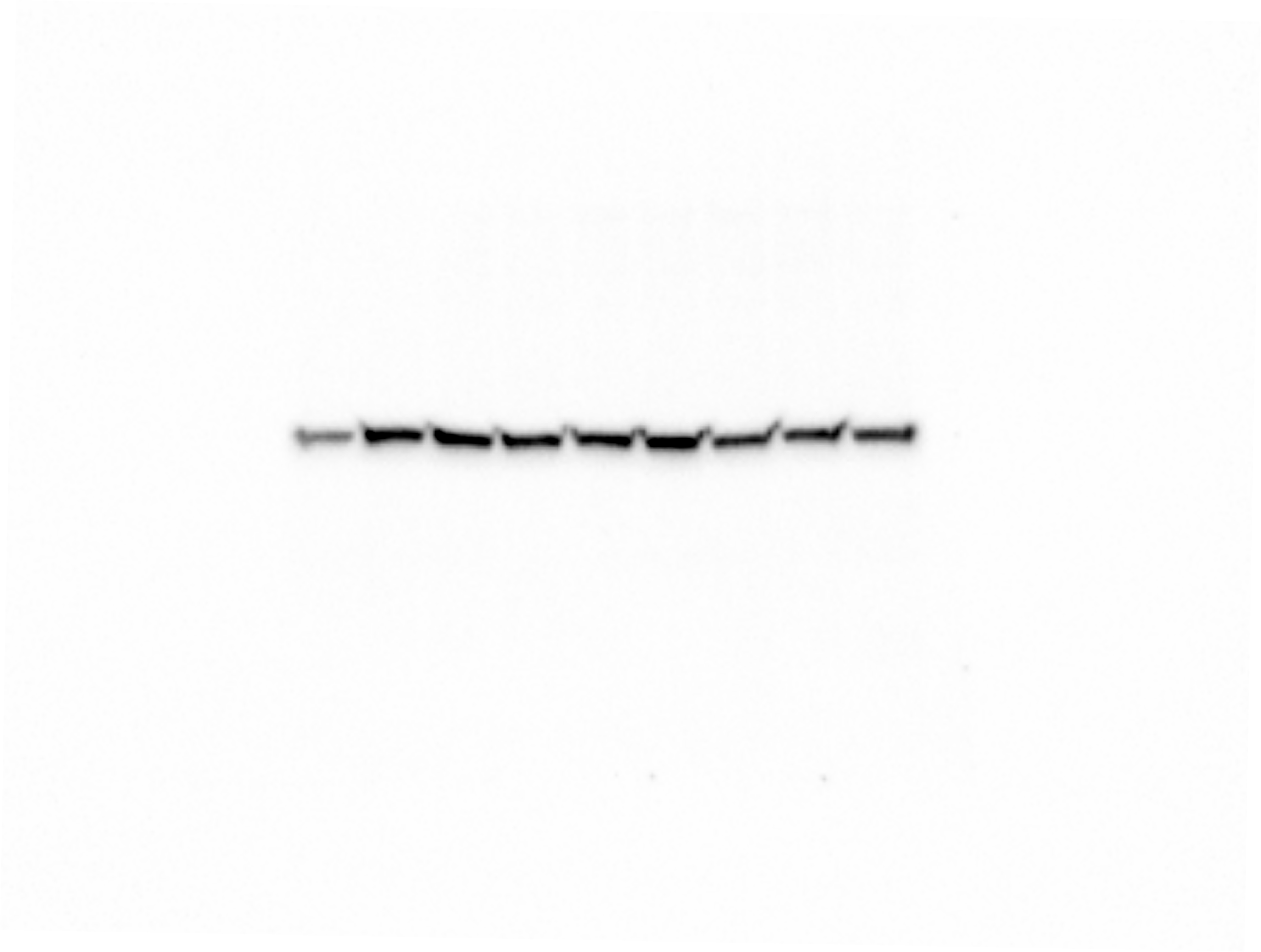

Supplement: Figure 3—figure supplement 2—source data 1. [file elife-84492-fig3-figsupp2-data1.zip › raw images/tubulin blot.tif]
