# Supplementary material for: Recruitment of Polo-like kinase couples synapsis to meiotic progression via inactivation of CHK-2
Source: eLife. 2023 Jan 26;12:e84492. doi: 10.7554/eLife.84492 (PMC9998088; doi:10.7554/eLife.84492)
Supplement: Supplementary file 1. — Supplementary file 1a reports the viability and fertility of representative transgenic worm strains used in this study, which indicates that all epitope- and degron-tagged alleles support normal meiosis and development. Supplementary file 1b lists the worm alleles generated in this study. Supplementary file 1c lists the crRNA, repair templates, and genotyping primers generated in this study. Supplementary file 1d lists the worm strains used in this study. [file elife-84492-supp1.docx]

**Supplementary file 1a**

**Viability and fertility of transgenic worm strains**

| Strains | Eggs laid (±SD)  (n=6 broods) | Egg viability (±SD) (%) | Male progeny (±SD) (%) |
| --- | --- | --- | --- |
| WT | 261(±17) | 106.64(±5.07) | 0.13(±0.30) |
| *meIs8[pie-1p::GFP::cosa-1, unc-119(+)] II; ieSi38[sun-1p::tir1::mRuby::sun-1 3’ UTR, Cbr-unc-119 (+)] IV;chk-2(ie121[HA::aid::chk-2]) V* | 295(±26.51) | 105.63(±3.09) | 0.27(±0.25) |
| *meIs8[pie-1p::GFP::cosa-1, unc-119(+)] II; ieSi38[sun-1p::tir1::mRuby::sun-1 3’ UTR, Cbr-unc-119 (+)] IV;chk-2(ie127[HA::chk-2]) V* | 202.33(±24.12) | 103.68(±3.64) | 0.15(±0.38) |
| *plk-2(ie50[plk-2::mRuby]; meIs8[pie-1p::GFP::cosa-1, unc-119(+)] II; mpk-1(ie54[mpk-1:: aid::3xFLAG]) III; ieSi38[sun-1p::tir1::mRuby::sun-1 3’ UTR, Cbr-unc-119 (+)] IV* | 274.6(±24.09) | 104.07(±3.11) | 0.15(±0.20) |
| *ieSi64[gld-1p::tir1::mRuby::gld-1 3’ UTR, Cbr-unc-119 (+)] II* | 269(±42.53) | 104.41(±1.88) | 0.07(±0.18) |
| *plk-2(ie126[plk-2::aid::HA]) I; ieSi64[gld-1p::tir1::mRuby::gld-1 3’ UTR, Cbr-unc-119 (+)] II; cosa-1(ie124[3xFLAG::cosa-1], plk-1(ie125[plk-1::aid::HA]) III* | 300.83(±40.66) | 101.02(±1.61) | 0.00(±0.00) |
| *meIs8[pie-1p::GFP::cosa-1, unc-119(+)] II; ieSi38[sun-1p::tir1::mRuby::sun-1 3’ UTR, Cbr-unc-119 (+)] IV; pas-1(ie185[pas-1::aid::3xFLAG]), chk-2(ie186 [ALFA::chk-2]) V* | 217.50(±33.75) | 103.57(±4.26) | 0.05(±0.14) |
| *plk-2(ie126[plk-2::aid::HA]) I; ieSi64[gld-1p::tir1::mRuby::gld-1 3’ UTR, Cbr-unc-119 (+)] II; cosa-1(ie124[3xFLAG::cosa-1], plk-1(ie125[plk-1::aid::HA]) III; pas-1(ie188[pas-1::aid::3xFLAG]), chk-2(ie187 [ALFA::chk-2]) V* | 245.50(±14.10) | 106.71(±5.17) | 0.00(±0.00) |

Note: viability >100% is a consequence of failing to count some embryos.

**Supplementary file 1b**

**Worm alleles generated in this study**

| Allele | Genotype | Information about mutagenesis |
| --- | --- | --- |
| *ie121* | *chk-2(ie121[HA::aid::chk-2])* | generated using *dpy-10* Co-CRISPR in *ieSi38[sun-1p::tir1::mRuby::sun-1 3'UTR, Cbr-unc-119(+)] IV* |
| *ie122* | *him-8 (ie122)* mutant | *generated using dpy-10 Co-CRISPR in meIs8[pie-1p::GFP::cosa-1, unc-119(+)] II; ieSi38[sun-1p::tir1::mRuby::sun-1 3'UTR, Cbr-unc-119(+)] IV; chk-2(ie121[HA::aid::chk-2]) V* |
| *ie123* | *syp-1(ie123[syp-1 T452A])* | *generated using dpy-10 Co-CRISPR in plk-2(ie50[plk-2::mRuby] I; meIs8[pie-1p::GFP::cosa-1, unc-119(+)] II* |
| *ieSi64* | *[gld-1p::tir1::mRuby::gld-1 3’ UTR, Cbr-unc-119 (+)] II* | Single copy transgene inserted into Chr II (oxTi179) using mosSCI |
| *ie124* | *cosa-1(ie124[3xFLAG::cosa-1]* | *generated using dpy-10 Co-CRISPR in ieSi64[gld-1p::tir1::mRuby::sun-1 3’ UTR, Cbr-unc-119 (+)] II* |
| *ie125* | *plk-1(ie125[plk-1::aid::HA])* | *generated using dpy-10 Co-CRISPR in ieSi64[gld-1p::tir1::mRuby::sun-1 3’ UTR, Cbr-unc-119 (+)] II; cosa-1(ie124[3xFLAG::cosa-1] III* |
| *ie126* | *plk-2(ie126[plk-2::aid::HA])* | *generated using dpy-10 Co-CRISPR in ieSi64[gld-1p::tir1::mRuby::sun-1 3’ UTR, Cbr-unc-119 (+)] II; cosa-1(ie124[3xFLAG::cosa-1] III* |
| *ie54* | *mpk-1(ie54[mpk-1::aid::3xFLAG])* | *generated using dpy-10 Co-CRISPR in plk-2(ie50[plk-2::mRuby] I; meIs8[pie-1p::GFP::cosa-1, unc-119(+)] II; ieSi38[sun-1p::tir1::mRuby::sun-1 3’ UTR, Cbr-unc-119 (+)] IV* |
| *ie127* | *chk-2(ie127[HA::chk-2])* | *generated using dpy-10 Co-CRISPR in meIs8[pie-1p::GFP::cosa-1, unc-119(+)] II; ieSi38[sun-1p::tir1::mRuby::sun-1 3’ UTR, Cbr-unc-119 (+)] IV* |
| *ie128* | *chk-2(ie128[HA::chk-2 S116A])* | *generated using dpy-10 Co-CRISPR in meIs8[pie-1p::GFP::cosa-1, unc-119(+)] II; ieSi38[sun-1p::tir1::mRuby::sun-1 3’ UTR, Cbr-unc-119 (+)] IV; chk-2(ie127[HA::chk-2])* |
| *ie129* | *chk-2(ie129[HA::chk-2 S116A])* | *generated using dpy-10 Co-CRISPR in meIs8[pie-1p::GFP::cosa-1, unc-119(+)] II; ieSi38[sun-1p::tir1::mRuby::sun-1 3’ UTR, Cbr-unc-119 (+)] IV; chk-2(ie127[HA::chk-2])* |
| *ie130* | *chk-2(ie130[HA::chk-2 S116D])* | *generated using dpy-10 Co-CRISPR in meIs8[pie-1p::GFP::cosa-1, unc-119(+)] II; ieSi38[sun-1p::tir1::mRuby::sun-1 3’ UTR, Cbr-unc-119 (+)] IV; chk-2(ie127[HA::chk-2])* |
| *ie131* | *chk-2(ie131[HA::chk-2 T120A])* | *generated using dpy-10 Co-CRISPR in meIs8[pie-1p::GFP::cosa-1, unc-119(+)] II; ieSi38[sun-1p::tir1::mRuby::sun-1 3’ UTR, Cbr-unc-119 (+)] IV; chk-2(ie127[HA::chk-2])* |
| *ie132* | *chk-2(ie132[HA::chk-2 T120A])* | *generated using dpy-10 Co-CRISPR in meIs8[pie-1p::GFP::cosa-1, unc-119(+)] II; ieSi38[sun-1p::tir1::mRuby::sun-1 3’ UTR, Cbr-unc-119 (+)] IV; chk-2(ie127[HA::chk-2])* |
| *ie133* | *chk-2(ie133[HA::chk-2 T120D])* | *generated using dpy-10 Co-CRISPR in meIs8[pie-1p::GFP::cosa-1, unc-119(+)] II; ieSi38[sun-1p::tir1::mRuby::sun-1 3’ UTR, Cbr-unc-119 (+)] IV; chk-2(ie127[HA::chk-2])* |
| *ie134* | *chk-2(ie134[HA::chk-2 T120D])* | *generated using dpy-10 Co-CRISPR in meIs8[pie-1p::GFP::cosa-1, unc-119(+)] II; ieSi38[sun-1p::tir1::mRuby::sun-1 3’ UTR, Cbr-unc-119 (+)] IV; chk-2(ie127[HA::chk-2])* |
| *ie135* | *plk-3(ie135) mutant* | *generated using dpy-10 Co-CRISPR in meIs8[pie-1p::GFP::cosa-1, unc-119(+)] II; ieSi38[sun-1p::tir1::mRuby::sun-1 3’ UTR, Cbr-unc-119 (+)], him-8(ie122) IV; chk-2(ie121[HA::aid::chk-2]) V* |
| *ie136* | *plk-3(ie136) mutant* | *generated using dpy-10 Co-CRISPR in plk-2(ie126[plk-2::aid::HA]) I; ieSi64[gld-1p::tir1::mRuby::sun-1 3’ UTR, Cbr-unc-119 (+)] II; cosa-1(ie124[3xFLAG::cosa-1], plk-1(ie125[plk-1::aid::HA]) III* |
| *ie185* | *pas-1(ie185[pas-1::aid::3xFLAG])* | *generated using dpy-10 Co-CRISPR in meIs8[pie-1p::GFP::cosa-1, unc-119(+)] II; ieSi38[sun-1p::tir1::mRuby::sun-1 3’ UTR, Cbr-unc-119 (+)] IV* |
| *ie186* | *chk-2(ie186[ALFA::chk-2])* | *generated using dpy-10 Co-CRISPR in meIs8[pie-1p::GFP::cosa-1, unc-119(+)] II; ieSi38[sun-1p::tir1::mRuby::sun-1 3’ UTR, Cbr-unc-119 (+)] IV; pas-1(ie185[pas-1::aid::3xFLAG]) V* |
| *ie187* | *chk-2(ie187[ALFA::chk-2])* | *generated using dpy-10 Co-CRISPR in plk-2(ie126[plk-2::aid::HA]) I; ieSi64[gld-1p::tir1::mRuby::gld-1 3’ UTR, Cbr-unc-119 (+)] II; cosa-1(ie124[3xFLAG::cosa-1], plk-1(ie125[plk-1::aid::HA]) III* |
| *ie188* | *pas-1(ie188[pas-1::aid::3xFLAG])* | *generated using dpy-10 Co-CRISPR in plk-2(ie126[plk-2::aid::HA]) I; ieSi64[gld-1p::tir1::mRuby::gld-1 3’ UTR, Cbr-unc-119 (+)] II; cosa-1(ie124[3xFLAG::cosa-1], plk-1(ie125[plk-1::aid::HA]) III; chk-2(ie187[ALFA::chk-2])V* |

**Supplementary file 1c**

**crRNAs, repair templates and genotyping primers used in this study**

| Transgenes | crRNAs and repair templates | Genotyping primers and fragment sizes |
| --- | --- | --- |
| *chk-2(ie121[HA::aid::chk-2])* | 5’-TCTTTTTGTCCCGCGAACCA-3’; 5’-attgaattttttgcgatttttggggcaaattttggtgattttttacTTTTCCGCCGAGCTCCGACGTCTcTTgGTtCCaCGgACTCCTGAGCCTCCcttcacgaacgccgccgcctccgggccaccgcttgatttttggcaggaaaccatcacgttcttccggtatgatctcaccggtggccatcccacaacttgtgccttggccggaggtttggctggatctttaggcatCGATCCGGCATAATCTGGCACATCATATGGGTACATggaaatcgctgaaaaaatggagaaaattgcgaaaaaatggagaaaattgcgaaaaaaacgaag- 3’ | F: 5’-ctacggtagtttttaaaggcgcag-3’; R: 5’-GATTCTCCGACGACAAGATCCTC-3’;  WT, 354bp; inserted, 534bp |
| *him-8 (ie122)* | 5’-TATCGACGTGCTCTCGGTGA-3’; NA | NA |
| *ieSi64 [gld-1p::tir1::mRuby::gld-1 3’ UTR, Cbr-unc-119 (+)] II* | NA; pLZ69 | oxTi179_II_F: 5’-TTGCCACGTCTTCTTGAGTG-3’; oxTi179_II_R: 5’-TGCTCGGAAGGACTTGATTT-3’.  WT, 281bp; inserted, >10kb |
| *cosa-1(ie124[3xFLAG::cosa-1])* | 5’-aagtgtcaATGTCAAGTTCT-3’; 5’-cgacaaaatcagtgaaaaatcgtgaaaactgaactgaagtgtcaATGGAtTAtAAaGACCAtGAtGGAGAcTAtAAGGAtCACGAtATtGAtTACAAaGACGAtGAtGAtAAaggagctggatcaTCAAGTTCTCGgtgagttgtcgtttcaaaataaaatgcgaacactgcctgtcaccatg- 3’ | F: 5’-cgtaaaccttcaaggcgcacag-3’; R: 5’-CTGATACGGCAGGTGTACCTAGAG-3’;  WT, 310bp; inserted, 388bp |
| *plk-1(ie125[plk-1::aid::HA])* | 5’-acatacggatCTATCGACGT-3’; 5’- GATATGCCACGGTCAATGGCAGCTGCTCGTTCAGCTTCGGCAGGATCACGAGGACCAAATCAAGCTGCATCGCACCTTCCCCAGTCGGCAAGTGGATCCAATATTCACCCACGTCGAggagctggatcaCCTAAAGATCCAGCCAAACCTCCGGCCAAGGCACAAGTTGTGGGATGGCCACCGGTGAGATCATACCGGAAGAACGTGATGGTTTCCTGCCAAAAATCAAGCGGTGGCCCGGAGGCGGCGGCGTTCGTGAAGggagccggatctTACCCCTACGATGTCCCAGATTATGCTTAGatccgtatgtgctctttgccacttccatgaatttgcttacatctcctgattaattccaagaagtattattctatacatttaacccgtactcttcatttattgttgctgtttttcatcgac- 3’ | F: 5’- cgtataatttacagAAATTCCTTCACCGAC-3’; R: 5’-catcagtatttacaatgaaatgagctacg-3’;  WT, 433bp; inserted, 616bp |
| *plk-2(ie126[plk-2::aid::HA])* | 5’-tcgattttcTTAGCGACGCG-3’; 5’-GAAAGAGGAGACGAAACACAATGCACCGGCGGCCAATGCAGTACGCCTTCCATCGACTTCCAGCAACGTCCGTTTGGAATCTGCAGCAGATATCCAGCCGGCTTATCCATCATCCTCGCGTCGCggagctggatcaCCTAAAGATCCAGCCAAACCTCCGGCCAAGGCACAAGTTGTGGGATGGCCACCGGTGAGATCATACCGGAAGAACGTGATGGTTTCCTGCCAAAAATCAAGCGGTGGCCCGGAGGCGGCGGCGTTCGTGAAGggagccggatctTACCCCTACGATGTCCCAGATTATGCTTAAgaaaatcgatctgcaacaaattgagctcatttccccttaccggttttgatatttctctgatcaatacacttttatgtccgtgtttgtaatcaattttatcc- 3’ | F: 5’-GGAGAAGTTCCTCCATCGAATTC-3’; R: 5’-gagcatgatgacacccgaatgtttg-3’;  WT, 451bp; inserted, 634bp |
| *mpk-1(ie54[mpk-1 aid::3xFLAG])* | 5’-TAAATACTACTAATCTAAAC-3’; 5’-GCGGCTGCAAGGAATAATGGAGGGCAGAATCCTGTTGGAGCTGGATCAATGCCTAAAGATCCAGCCAAACCTCCGGCCAAGGCACAAGTTGTGGGATGGCCACCGGTGAGATCTTACCGGAAGAACGTGATGGTTTCCTGCCAAAAATCAAGCGGTGGCCCGGAGGCGGCGGCGTTCGTGAAGGGAGCCGGATCTGATTATAAAGACCATGATGGAGACTATAAGGATCACGATATTGATTACAAAGACGATGATGATAAATAATAGATTAGTAGTATTTACCCACTAAATTAGTTATTTTTTCCACTTTTTTTTTATTTTCCACTAAGATTTTGGCATTTCAGTTTCTTTTTCGATGTATCATAATCCACTTCAAAACTCGATCG- 3’ | F: 5’- CAGTTTGTGAGGAACCATTCACTTTGG -3’; R: 5’-gagtgattaattggaggaggtgcattc-3’;  WT, 426bp; inserted, 654bp |
| *chk-2(ie127[HA::chk-2])* | 5’-tcagcgatttccATGGTTCG-3’; 5’-ccattttttcgcaattttctccattttttcagcgatttccATGTACCCATATGATGTGCCAGATTATGCCGGAGGCTCAGGAGTTCGCGGGACAAAAAGACGTCGGAGCTCGGCGGAAAAgtaaaaaatcac- 3’ | F: 5’-gtctcgccgcgattttcgtattttc-3’; R: 5’-CAAGCTTCGCGAAGGGTTTTGAAG-3’;  WT, 339bp; inserted, 378bp |
| *chk-2(ie128[HA::chk-2 S116A]) and chk-2(ie129[HA::chk-2 S116A])* | 5’-TACACGATCACTCACGCAAC-3’; 5’-caatatttcagGACACAGAAACGCGTAGAATCTATCTACACGATCACgCcCGCAACGGaACCCTCGTAAATCAGGAAATGATCGGAAAAGGGCTGTCCAGAGAG- 3’ | F: 5’-gattttccagACTTGGCAGACGATC-3’; R: 5’-cgtcaaaaacgcacTGATCAGCGCTG-3’;  WT, 337bp; mutant, 337bp=223bp+114bp (Cac8I digestion) |
| *chk-2(ie130[HA::chk-2 S116D])* | 5’-TACACGATCACTCACGCAAC-3’; 5’-caatatttcagGACACAGAAACGCGTAGAATCTATCTACACGATCACgatCGCAACGGaACCCTCGTAAATCAGGAAATGATCGGAAAAGGGCTGTCCAGAGAG- 3’ | F: 5’-gattttccagACTTGGCAGACGATC-3’; R: 5’-cgtcaaaaacgcacTGATCAGCGCTG-3’;  WT, 337bp; mutant, 337bp=223bp+114bp (PvuI digestion) |
| *chk-2(ie131[HA::chk-2 T120A]) and chk-2(ie132[HA::chk-2 T120A])* | 5’-CGATCATTTCCTGATTTACG-3’; 5’- CTCCGTTCATTAGCTCTCTGGACAGCCCTTTTCCGATCATTTCCTGATTTACGAGaGctCCGTTGCGTGAGTGATCGTGTAGATAGATTCTACGCGTTTCTGTGTCctgaaatattg- 3’ | F: 5’-gattttccagACTTGGCAGACGATC-3’; R: 5’-cgtcaaaaacgcacTGATCAGCGCTG-3’;  WT, 337bp; mutant, 337bp=233bp+104bp (SacI digestion) |
| *chk-2(ie133[HA::chk-2 T120D]) and chk-2(ie134[HA::chk-2 T120D])* | 5’-CGATCATTTCCTGATTTACG-3’; 5’- CTCTCTGGACAGCCCTTTTCCGATCATTTCCTGATTTACGAGatcCCCGTTGCGTGAGTGATCGTGTAGATAGATTCTACGCGTTTCTGTGTCctgaaatattg- 3’ | F: 5’-gattttccagACTTGGCAGACGATC-3’; R: 5’-cgtcaaaaacgcacTGATCAGCGCTG-3’;  WT, 337bp; mutant, 337bp=234bp+103bp (XhoII digestion) |
| *plk-3 (ie135) and plk-3 (ie136)* | 5’-GCTCCGTACTCGCGGAAACT-3’; 5’- caaatttattcatcgtttctagATGCAGCATGTGCTCCGTACTCGCGGAAACTAAGCTCGAGTCGGCACAAGATAAAAATAAAAAGCATGTTCCTAATGTACCGCCAATTATCTAC- 3’ | F: 5’-caacgtgtttagttgaattgcacttc-3’; R: 5’- CTGTCGATGAATTTCAACTTCTCGAG-3’;  WT, 380bp; mutant, 390bp=150bp+240bp (XhoI digestion) |
| *pas-1(ie185[pas-1::aid::3xFLAG]) and pas-1(ie188[pas-1::aid::3xFLAG])* | 5’- gatgaatatTTAATCTCGGT -3’; 5’- CAAGGATCTTGAGGTTGTCGTTGTCACCAAGGATAACTCCAAATTCACCAAGCTAACCAGCGATCAGGTCGAACATCATCTCAATCAAATCGCCAACCGAGATggagctggatcaCCTAAAGATCCAGCCAAACCTCCGGCCAAGGCACAAGTTGTGGGATGGCCACCGGTGAGATCATACCGGAAGAACGTGATGGTTTCCTGCCAAAAATCAAGCGGTGGCCCGGAGGCGGCGGCGTTCGTGAAGggagccggatctGAtTAtAAaGACCAtGAtGGAGAcTAtAAGGAtCACGAtATtGAtTACAAaGACGAtGAtGAtAAaTAAatattcatcgacctttgtatttaaatatttcccgtttcaattgatccggtacccaaattgaattaaatacctggtttcc -3’ | F: 5’- CAACTTCCAGCGACCTCATTCTTG -3’; R: 5’- ggagggcaatgataaaaaagacc -3’;  WT, 373bp; inserted, 595bp |
| *chk-2(ie186[ALFA::chk-2]) and chk-2(ie187[ALFA::chk-2])* | 5’- tcagcgatttccATGGTTCG -3’; 5’- ccattttttcgcaattttctccattttttcagcgatttccATGCCATCCCGTCTCGAGGAGGAGCTCCGTCGTCGTCTCACCGAGCCAGGAGGCTCAGGAGTTCGCGGGACAAAAAGACGTCGGAGCTCGGCGGAAAAgtaaaaaatcac -3’ | F: 5’- gtctcgccgcgattttcgtattttc -3’; R: 5’- CAAGCTTCGCGAAGGGTTTTGAAG -3’;  WT, 339bp; inserted, 396bp |

**Supplementary file 1d**

**Worm strains used in this study**

| Strains | Source | Identifier |
| --- | --- | --- |
| *C. elegans:* N2 *Bristol, wild isolate* | Caenorhabditis Genetics Center | N2 |
| *C. elegans: plk-2(ie50[plk-2::mRuby] I; meIs8[pie-1p::GFP::cosa-1, unc-119(+)] II; syp-1(ie123[syp-1 T452A]) V* | This paper | CA1290 |
| *C. elegans: ieSi64[gld-1p::tir1::mRuby::gld-1 3’ UTR, Cbr-unc-119 (+)] II* | This paper | CA1352 |
| *C. elegans: meIs8[pie-1p::GFP::cosa-1, unc-119(+)] II; ieSi38[sun-1p::tir1::mRuby::sun-1 3’ UTR, Cbr-unc-119 (+)] IV* | This paper | CA1364 |
| *C. elegans: plk-2(ie50[plk-2::mRuby] I; meIs8[pie-1p::GFP::cosa-1, unc-119(+)] II; mpk-1(ie54[mpk-1::aid::3xFLAG]) III; ieSi38[sun-1p::tir1::mRuby::sun-1 3’ UTR, Cbr-unc-119 (+)] IV* | This paper | CA1416 |
| *C. elegans: plk-2(ie108[plk-2::aid::3xFLAG]), zhp-2(ie107[zhp-2::HA]) I; meIs8[pie-1p::GFP::cosa-1, unc-119(+)] II; ieSi38[sun-1p::tir1::mRuby::sun-1 3'UTR, Cbr-unc-119(+)] IV* | Zhang et al., 2018 | CA1429 |
| *C. elegans: meIs8[pie-1p::GFP::cosa-1, unc-119(+)] II; ieSi38[sun-1p::tir1::mRuby::sun-1 3’ UTR, Cbr-unc-119 (+)] IV; chk-2(ie121[HA::aid::chk-2]) V* | This paper | CA1539 |
| *C. elegans: meIs8[pie-1p::GFP::cosa-1, unc-119(+)] II; him-8(ie122), ieSi38[sun-1p::tir1::mRuby::sun-1 3’ UTR, Cbr-unc-119 (+)] IV; chk-2(ie121[HA::aid::chk-2]) V* | This paper | CA1540 |
| *C. elegans: plk-2(tm1395) I; meIs8[pie-1p::GFP::cosa-1, unc-119(+)] II; ieSi38[sun-1p::tir1::mRuby::sun-1 3’ UTR, Cbr-unc-119 (+)] IV; chk-2(ie121[HA::aid::chk-2]) V* | This paper | CA1541 |
| *C. elegans: plk-2(ie50[plk-2::mRuby] I??; meIs8[pie-1p::GFP::cosa-1, unc-119(+)] II; ieSi38[sun-1p::tir1::mRuby::sun-1 3’ UTR, Cbr-unc-119 (+)] IV; syp-1(ie123[syp-1 T452A]), chk-2(ie121[HA::aid::chk-2]) V* | This paper | CA1542 |
| *C. elegans: plk-2(ie126[plk-2::aid::HA]) I; ieSi64[gld-1p::tir1::mRuby::gld-1 3’ UTR, Cbr-unc-119 (+)] II; cosa-1(ie124[3xFLAG::cosa-1], plk-1(ie125[plk-1::aid::HA]) III* | This paper | CA1543 |
| *C. elegans: plk-2(ie50[plk-2::mRuby] I; meIs8[pie-1p::GFP::cosa-1, unc-119(+)] II; mpk-1(ie54[mpk-1::aid::3xFLAG]) III; ieSi38[sun-1p::tir1::mRuby::sun-1 3’ UTR, Cbr-unc-119 (+)] IV; syp-1(ie123[syp-1 T452A]) V* | This paper | CA1544 |
| *C. elegans: ieSi64[gld-1p::tir1::mRuby::gld-1 3’ UTR, Cbr-unc-119 (+)] II; cosa-1(ie124[3xFLAG::cosa-1] III* | This paper | CA1545 |
| *C. elegans: meIs8[pie-1p::GFP::cosa-1, unc-119(+)] II; ieSi38[sun-1p::tir1::mRuby::sun-1 3’ UTR, Cbr-unc-119 (+)] IV; chk-2(ie127[HA::chk-2]) V* | This paper | CA1546 |
| *C. elegans: plk-2(ie50[plk-2::mRuby] I??; meIs8[pie-1p::GFP::cosa-1, unc-119(+)] II; ieSi38[sun-1p::tir1::mRuby::sun-1 3’ UTR, Cbr-unc-119 (+)] IV??; syp-1(ie123[syp-1 T452A]), chk-2(ie127[HA::chk-2]) V* | This paper | CA1547 |
| *C. elegans: meIs8[pie-1p::GFP::cosa-1, unc-119(+)] II; ieSi38[sun-1p::tir1::mRuby::sun-1 3’ UTR, Cbr-unc-119 (+)] IV; chk-2(ie128[HA::chk-2 S116A]) V* | This paper | CA1548 |
| *C. elegans: meIs8[pie-1p::GFP::cosa-1, unc-119(+)] II; ieSi38[sun-1p::tir1::mRuby::sun-1 3’ UTR, Cbr-unc-119 (+)] IV; chk-2(ie129[HA::chk-2 S116A])WT V* | This paper | CA1549 |
| *C. elegans: meIs8[pie-1p::GFP::cosa-1, unc-119(+)] II; ieSi38[sun-1p::tir1::mRuby::sun-1 3’ UTR, Cbr-unc-119 (+)] IV; chk-2(ie130[HA::chk-2 S116D])/WT V* | This paper | CA1550 |
| *C. elegans: meIs8[pie-1p::GFP::cosa-1, unc-119(+)] II; ieSi38[sun-1p::tir1::mRuby::sun-1 3’ UTR, Cbr-unc-119 (+)] IV; chk-2(ie131[HA::chk-2 T120A])/WT V* | This paper | CA1551 |
| *C. elegans: meIs8[pie-1p::GFP::cosa-1, unc-119(+)] II; ieSi38[sun-1p::tir1::mRuby::sun-1 3’ UTR, Cbr-unc-119 (+)] IV; chk-2(ie132[HA::chk-2 T120A])/WT V* | This paper | CA1552 |
| *C. elegans: meIs8[pie-1p::GFP::cosa-1, unc-119(+)] II; ieSi38[sun-1p::tir1::mRuby::sun-1 3’ UTR, Cbr-unc-119 (+)] IV; chk-2(ie133[HA::chk-2 T120D])/WT V* | This paper | CA1553 |
| *C. elegans: meIs8[pie-1p::GFP::cosa-1, unc-119(+)] II; ieSi38[sun-1p::tir1::mRuby::sun-1 3’ UTR, Cbr-unc-119 (+)] IV; chk-2(ie134[HA::chk-2 T120D])/WT V* | This paper | CA1554 |
| *C. elegans: meIs8[pie-1p::GFP::cosa-1, unc-119(+)] II; ieSi38[sun-1p::tir1::mRuby::sun-1 3’ UTR, Cbr-unc-119 (+)], plk-3(ie135), him-8(ie122) IV; chk-2(ie121[HA::aid::chk-2]) V* | This paper | CA1555 |
| *C. elegans: plk-2(ie126[plk-2::aid::HA]) I; ieSi64[gld-1p::tir1::mRuby::gld-1 3’ UTR, Cbr-unc-119 (+)] II; cosa-1(ie124[3xFLAG::cosa-1], plk-1(ie125[plk-1::aid::HA]) III; plk-3(ie136) IV* | This paper | CA1556 |
| *C. elegans: meIs8[pie-1p::GFP::cosa-1, unc-119(+)] II; ieSi38[sun-1p::tir1::mRuby::sun-1 3’ UTR, Cbr-unc-119 (+)] IV; him-5(ok1896), chk-2(ie121 [HA::aid::chk-2]) V* | This paper | CA1557 |
| *C. elegans: meIs8[pie-1p::GFP::cosa-1, unc-119(+)] II; ieSi38[sun-1p::tir1::mRuby::sun-1 3’ UTR, Cbr-unc-119 (+)] IV; pas-1(ie185[pas-1::aid::3xFLAG]) V* | This paper | CA1558 |
| *C. elegans: meIs8[pie-1p::GFP::cosa-1, unc-119(+)] II; ieSi38[sun-1p::tir1::mRuby::sun-1 3’ UTR, Cbr-unc-119 (+)] IV; pas-1(ie185[pas-1::aid::3xFLAG]), chk-2(ie186 [ALFA::chk-2]) V* | This paper | CA1559 |
| *C. elegans: plk-2(ie126[plk-2::aid::HA]) I; ieSi64[gld-1p::tir1::mRuby::gld-1 3’ UTR, Cbr-unc-119 (+)] II; cosa-1(ie124[3xFLAG::cosa-1], plk-1(ie125[plk-1::aid::HA]) III; chk-2(ie187 [ALFA::chk-2]) V* | This paper | CA1628 |
| *C. elegans: plk-2(ie126[plk-2::aid::HA]) I; ieSi64[gld-1p::tir1::mRuby::gld-1 3’ UTR, Cbr-unc-119 (+)] II; cosa-1(ie124[3xFLAG::cosa-1], plk-1(ie125[plk-1::aid::HA]) III; pas-1(ie188[pas-1::aid::3xFLAG]), chk-2(ie187 [ALFA::chk-2]) V* | This paper | CA1629 |
